# Case Report: Severe protein S deficiency unmasks a cryptic *PROC* mutation with normal activity, triggering life-threatening pulmonary thromboembolism

**DOI:** 10.3389/fcvm.2026.1804812

**Published:** 2026-04-13

**Authors:** Yafei Liu, Binbin Zhou, Jianjian Xue, Wei Zhang, Jiewei Liu

**Affiliations:** 1 Department of Emergency Medicine, Fuzong Clinical Medical College of Fujian Medical University, Fuzhou, Fujian, China; 2 Department of Emergency, 900th Hospital of PLA Joint Logistic Support Force, Fuzhou, Fujian, China; 3 Department of Emergency Medicine, Fuzong Teaching Hospital of Fujian University of Traditional Chinese Medicine (900th Hospital), Fuzhou, Fujian, China

**Keywords:** ECMO, inherited thrombophilia, interventional thrombectomy, *PROC*, *PROS1*, pulmonary thromboembolism

## Abstract

**Background:**

In young patients presenting with high-risk, idiopathic pulmonary thromboembolism (PTE), prompt identification of the underlying prothrombotic state is critical. Inherited deficiencies of protein C (PC) and protein S (PS) are predominant risk factors in Asians. However, routine functional assays for PC/PS activity may not capture the full genetic risk. Specifically, the clinical significance of a pathogenic *PROC* mutation in individuals with normal PC activity remains poorly defined. We hypothesize that in such individuals, the thrombogenic potential of a “cryptic” *PROC* defect may remain latent until unmasked by a profound cofactor deficiency, such as severe PS deficiency. This synergistic mechanism, and the catastrophic thrombosis it may precipitate, has not been previously documented.

**Case presentation:**

A previously healthy 20-year-old male presented with sudden-onset high-risk pulmonary thromboembolism and obstructive shock. Despite systemic thrombolysis, he progressed to refractory shock and was successfully rescued with VA-ECMO bridging to percutaneous mechanical thrombectomy. Coagulation workup revealed severely reduced PS activity (28.3%); notably, protein C activity was normal at 99.7%. Genetic testing identified heterozygous pathogenic mutations in *PROS1* (c.1680T > A, p.Tyr560Ter) and *PROC* (c.577_579delAAG, p.Lys193del), inherited from his asymptomatic parents respectively.

**Conclusions:**

This case demonstrates that severe PS deficiency can unmask the thrombogenic potential of a pathogenic *PROC* mutation even in the setting of normal PC activity, revealing a previously underrecognized synergistic prothrombotic mechanism. It underscores the critical importance of systematic thrombophilia screening, including genetic testing for *PROS1* and *PROC*, in young patients with high-risk PTE even when PC activity is normal.

## Keypoints

- First report of severe protein S deficiency unmasking a pathogenic *PROC* mutation despite normal PC activity, triggering life-threatening PTE.
- Proposes a novel “two-hit” synergistic mechanism underlying catastrophic thrombosis.
- Highlights the critical role of VA-ECMO-bridged thrombectomy in refractory shock.
- Advocates for genetic testing in young high-risk PE patients even if PC activity is normal.

## 1 Introduction

High-risk pulmonary thromboembolism (PTE) carries a high mortality, necessitating immediate reperfusion and hemodynamic support (1). In young patients presenting with high-risk PTE, identifying the underlying prothrombotic state is paramount for guiding acute management and long-term secondary prevention. Inherited thrombophilia represents a major intrinsic risk factor, with a distinct genetic landscape across ethnicities (2). Notably, while the predominant genetic risk in Western populations stems from gain-of-function mutations (*Factor V Leiden* and *prothrombin G20210A*) (3), in East Asian populations, deficiencies of protein C (PC) and protein S (PS) constitute the major etiology, with *PROS1* and *PROC* mutations accounting for a substantial proportion of familial venous thromboembolism cases (4, 5).

Consequently, functional assays of PC and PS activity are routinely employed in the thrombophilia workup of young Asian patients with PTE. However, this screening paradigm may harbor a critical blind spot. The specific *PROC* mutation (c.577_579delAAG, p.Lys193del) identified in our patient has been reported as a recurrent risk variant, typically in association with reduced PC activity (6). This raises an unanswered question: what is the clinical risk when such a pathogenic *PROC* variant is present alongside normal PC activity? We hypothesize that in this setting, the thrombogenic potential of the *PROC* defect may remain clinically silent—a “cryptic” risk—until unmasked by a profound cofactor deficiency. Given that PS is an essential cofactor for activated PC function (7), severe PS deficiency could critically impair the entire anticoagulant pathway, thereby unleashing the latent threat of the coexisting *PROC* mutation. This postulated synergistic mechanism for catastrophic thrombosis remains undocumented, and the optimal management strategy for PTE arising from such a complex genetic background is unclear.

To address these questions, we report the first case of life-threatening PTE in a young male, where severe PS deficiency unmasked the thrombogenic potential of a pathogenic *PROC* mutation despite normal circulating PC activity. This case was successfully rescued with an integrated strategy centered on VA-ECMO-bridged interventional thrombectomy. Herein, we describe this novel synergistic prothrombotic mechanism, highlight the imperative to incorporate genetic testing into the diagnostic algorithm for high-risk PTE, and discuss the multidisciplinary management approach.

## 2 Case presentation

A previously healthy 20-year-old male presented with sudden-onset dyspnea, epigastric pain, and diaphoresis, which progressed to loss of consciousness during transfer. On admission, he was in obstructive shock with unrecordable blood pressure and oxygen saturation. Despite emergent intubation, aggressive fluid resuscitation, and high-dose vasopressor support, his systolic blood pressure remained critically low at approximately 80 mmHg. A detailed history obtained from his family revealed a sedentary lifestyle with prolonged daily sitting due to his occupation as a computer science student, but no personal or family history of thrombosis, recent trauma, surgery, immobilization, infection (including COVID-19), or other acquired thrombotic risk factors.

**Figure 1:**
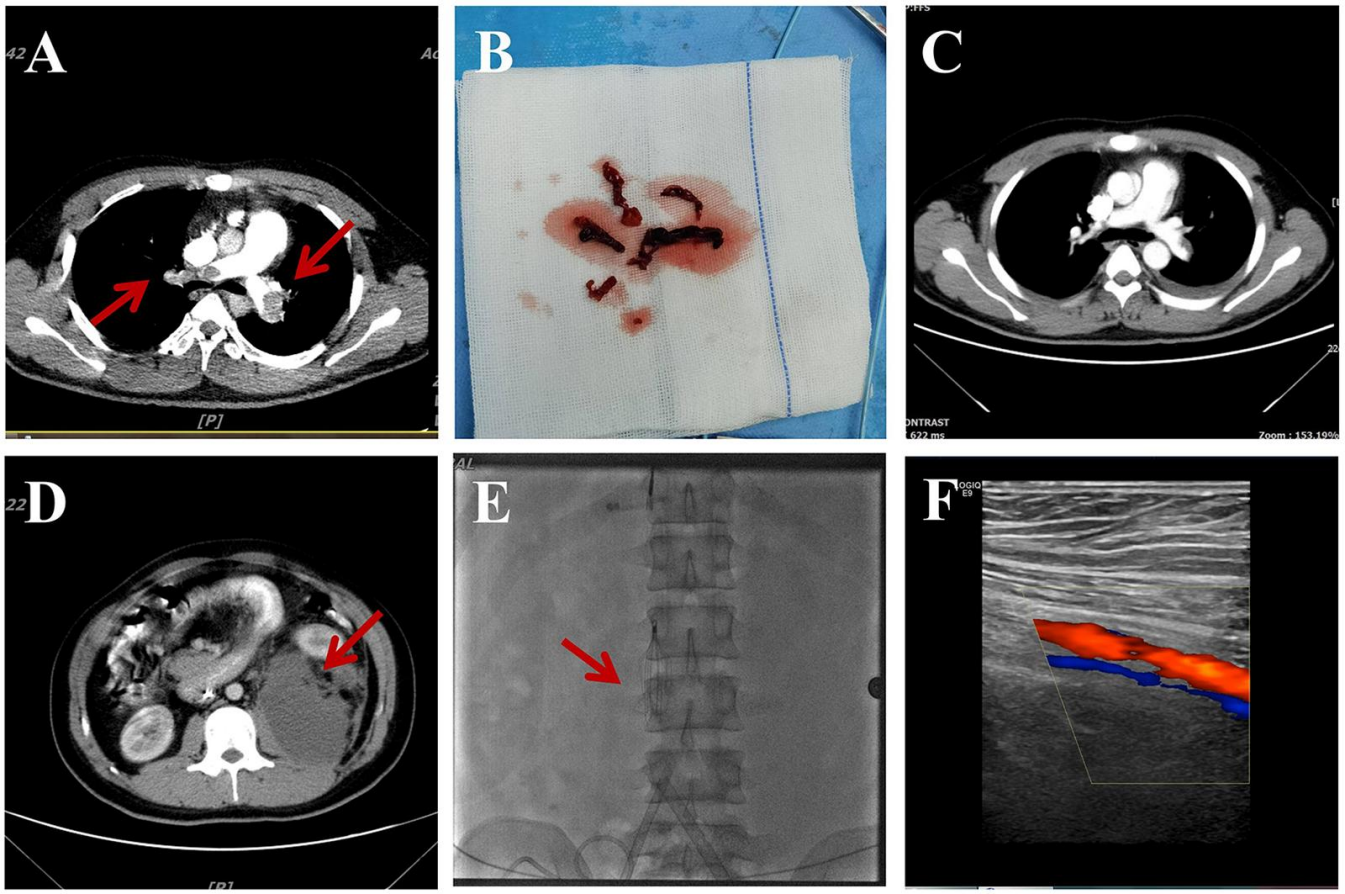
Key clinical imaging and pathological findings of the patient. **(A)** CT pulmonary angiography on admission showing bilateral pulmonary embolism (Red arrows). **(B)** Thrombus specimen retrieved by ECMO-assisted interventional thrombectomy. **(C)** Angiography after thrombectomy showing restored pulmonary blood flow. **(D)** Abdominal CT demonstrating a retroperitoneal hematoma (Red arrow). **(E)** Post-procedural imaging after inferior vena cava filter placement (Red arrow). **(F)** Six-month follow-up venous ultrasound of the left lower extremity showing chronic thrombosis.

Initial laboratory investigation revealed severe acidosis and end-organ hypoperfusion (pH 7.24, lactate 9.8 mmol/L) alongside markedly elevated biomarkers of myocardial strain and thrombosis (high-sensitivity troponin T 6.170 ng/mL, NT-proBNP 13507 pg/mL, D-dimer 6.70 mg/L FEU). CT pulmonary angiography confirmed extensive bilateral proximal pulmonary artery emboli (Figure 1A), and bedside echocardiography demonstrated acute cor pulmonale with right ventricular dilation (RV/LV ratio >1) and severely reduced left ventricular ejection fraction (30%).

Despite the administration of systemic thrombolysis with alteplase (100 mg) within two hours of admission, the patient deteriorated into refractory shock with hemorrhagic complications, indicating failure of first-line reperfusion therapy. As a lifesaving measure, veno-arterial extracorporeal membrane oxygenation (VA-ECMO) was emergently initiated to provide hemodynamic and respiratory support. This was immediately bridged to percutaneous mechanical thrombectomy, which successfully restored pulmonary blood flow (Figures 1B,C). The subsequent clinical course was complicated by acute kidney injury requiring continuous renal replacement therapy and a significant bleeding risk that necessitated the use of nafamostat mesylate for anticoagulation.

Once hemodynamically stabilized, a thorough etiological workup for catastrophic thrombosis was pursued. Crucially, coagulation studies revealed a stark dissociation: PS activity was severely deficient at 28.3% (reference range 60%–130%), while PC activity was unequivocally normal at 99.7% (reference range 70%–150%). All other tests for inherited and acquired thrombophilia (including antithrombin III, antiphospholipid antibodies) were unremarkable.

**Figure 2:**
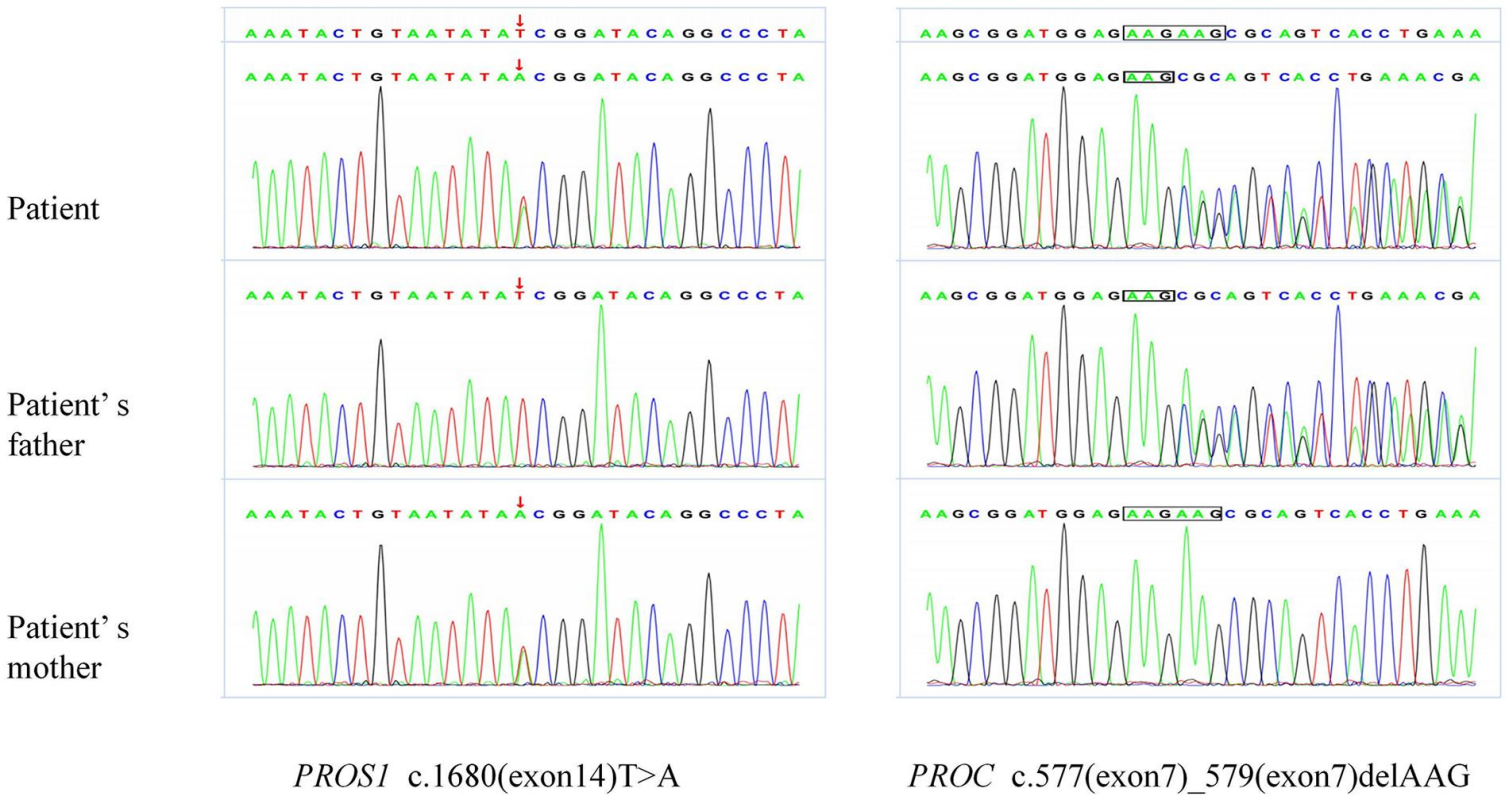
Genetic sequencing analysis of the patient and his parents. Left panel (*PROS1* c.1680T > A): The nonsense mutation (p.Tyr560Ter) in exon 14 is indicated by arrows. Right panel (*PROC* c.577_579delAAG): The in-frame deletion (p.Lys193del) in exon 7 is marked by boxes. Segregation analysis confirmed maternal inheritance of the *PROS1* variant and paternal inheritance of the *PROC* variant.

To unravel this paradox, comprehensive genetic testing was performed. It identified heterozygous pathogenic mutations in two key genes: a nonsense mutation in *PROS1* (c.1680T > A, p.Tyr560Ter) and an in-frame deletion in *PROC* (c.577_579delAAG, p.Lys193del). Familial segregation analysis provided the definitive link: the *PROS1* mutation was inherited from his mother (who had reduced PS activity of 10%), and the *PROC* mutation from his father (a carrier with normal PC/PS activity) (Figure 2). This confirmed the *de novo* confluence of two distinct genetic defects in the proband.

The patient's hospitalization was prolonged due to a large retroperitoneal hematoma at the ECMO cannulation site (Figure 1D), which was managed conservatively. Immediately prior to successful ECMO decannulation on day 4, an inferior vena cava filter was placed (Figure 1E) due to documented deep vein thrombosis of the lower extremities; this filter was later retrieved after three months. He was discharged on day 30 on long-term therapeutic anticoagulation with rivaroxaban. Follow-up at six months showed residual chronic thrombosis on ultrasound (Figure 1F), requiring careful dose adjustment. A summarized timeline is provided in Table 1.

**Table 1:** Clinical timeline of the case.

Time point (Day from admission)	Key clinical events	Clinical Stage	Associated figures
0	Symptom onset: sudden dyspnea, epigastric pain, diaphoresis, progressing to altered consciousness during transfer	Presentation	–
0	Admission with obstructive shock with unrecordable BP/O₂ saturation; emergent intubation, fluid resuscitation, high-dose vasopressors (SBP ≈60 mmHg)	Critical care	–
0	Diagnostics: pH 7.24, lactate 9.8 mmol/L; elevated D-dimer (6.70 mg/L), hs-TnT (6.170 ng/mL), NT-proBNP (13507 pg/mL)	Laboratory	–
0	Imaging: CTPA confirmed bilateral proximal pulmonary artery filling defects; bedside echo showed RV dilation (RV/LV >1), LVEF 30%	Diagnostic	Figure 1A
0	Treatment: Systemic thrombolysis with alteplase (100 mg) within 2 h of admission	Pharmacological	–
0	Complication: Progression to refractory shock with hemorrhagic airway secretions	Complication	–
0	Rescue: Emergency VA-ECMO initiation followed by pulmonary artery thrombectomy	Procedural	Figures 1B,C
0–4	Concurrent acute kidney injury and hyperkalemia; continuous renal replacement therapy initiated; anticoagulation switched to nafamostat mesylate due to bleeding risk	Supportive care	–
4	Deep vein thrombosis confirmed by lower extremity ultrasound; inferior vena cava filter placed	Procedural	Figure 1E
4	Successful ECMO decannulation	Milestone	–
13	Extubation	Milestone	–
30	Significant resolution of pulmonary embolism, retroperitoneal hematoma (9.9 × 8.6 cm) noted and managed conservatively. Discharge in stable condition; long-term anticoagulation with rivaroxaban 30 mg once daily (reduced to 20 mg after 3 weeks);	Discharge	Figure 1D
90	3-month follow-up: Significant resolution of pulmonary embolism; residual lower-extremity thrombosis led to filter retrieval; retroperitoneal hematoma absorbed (5.3 × 3.8 cm)	Follow-up	–
180	6-month follow-up: Ultrasound suggested chronic thrombosis in left lower extremity; elevated D-dimer (1.0 mg/L) prompted temporary increase of rivaroxaban to 30 mg daily for 3 weeks, then back to 20 mg maintenance	Follow-up	Figure 1F

## 3 Discussion

### 3.1 A novel “two-hit” unmasking mechanism

To our knowledge, this is the first report to demonstrate that severe PS deficiency can unmask the life-threatening thrombogenic potential of a pathogenic *PROC* mutation despite normal circulating PC activity. We propose this occurs through a novel “two-hit” synergistic mechanism, which we delineate as follows.

#### 3.1.1 The first hit: A cryptic qualitative defect in PC

The *PROC* p.Lys193del variant deletes a lysine residue within the epidermal growth factor-2 (EGF2) domain, a region critical for PC activation. This mutation is known to spare the amidolytic activity of PC, directly accounting for the patient's normal plasma PC activity (99.7%). This finding challenges the conventional paradigm that pathogenic *PROC* mutations invariably reduce measurable PC activity (6). We propose that the variant instead confers a cryptic qualitative defect: it preserves basal enzymatic function while compromising physiological anticoagulant activity. This hypothesis is supported by functional studies showing that the same mutation impairs clot-based anticoagulant activity and increases factor V activity (8). Thus, the p.Lys193del variant acted as a “silent” first genetic hit, establishing a latent prothrombotic state that remained clinically dormant until unmasked.

#### 3.1.2 The second hit: profound PS deficiency disabling the pathway

The critical precipitating factor was severe PS deficiency (activity 28.3%). PS is the indispensable cofactor for APC (9). Its profound loss creates a critical bottleneck, disabling the entire PC anticoagulant pathway. Even with normal levels of PC, the resulting APC cannot efficiently assemble the functional complex on cell membranes required to inactivate factors Va and VIIIa (10). Foundational studies confirm that complete disruption of this pathway leads to a lethal prothrombotic state in models (11). Therefore, severe PS deficiency constituted the second hit, generating a systemically disabled anticoagulant environment.

#### 3.1.3 Synergistic unmasking: the confluence of hits

The catastrophic thrombosis arose from the synergistic confluence of these two hits. The profound PS deficiency (second hit) created a disabled pathway background- a necessary but insufficient condition. Within this disabled state, the latent qualitative defect of the *PROC* mutation (first hit) was catastrophically unmasked. The synergy is not merely additive but fundamental: one hit disables the system, while the other supplies a flawed component within it, culminating in complete regulatory failure and fulminant thrombosis. This mechanism elegantly explains the stark phenotypic contrast with the asymptomatic parents, each carrying only one isolated defect.

### 3.2 Contrast with existing literature and validation of novelty

Our case defines a novel, catastrophic phenotype that extends the known spectrum of *PROC*/*PROS1* double heterozygosity. Previous studies, primarily in East Asian cohorts, establish that co-inheritance of these mutations confers a significant thrombotic risk, typically presenting as provoked deep vein thrombosis (DVT) accompanied by concordant reductions in both PC and PS activity-a consistent genotype-phenotype correlation (6, 12–15).

In stark contrast, our proband exhibited a life-threatening, high-risk PTE with refractory shock, representing the most severe extreme of the clinical spectrum. Crucially, laboratory evaluation revealed a critical dissociation: severely reduced PS activity (28.3%) alongside unequivocally normal PC activity (99.7%). This observation highlights two pivotal advances:

Clinical Severity: This is the first reported case where double *PROC*/*PROS1* heterozygosity precipitated immediately life-threatening PTE requiring VA-ECMO rescue, moving beyond the previously reported context of (usually provoked) DVT.

Laboratory Phenotype: We document the first instance of this genetic combination presenting with normal PC activity, thereby breaking the established link between the pathogenic *PROC* genotype and its expected functional deficit.

Therefore, our case reveals a previously unrecognized high-risk clinico-pathophysiological entity: catastrophic thrombosis driven by the synergistic unmasking of a cryptic *PROC* defect under conditions of severe-not merely moderate-PS deficiency. This finding challenges the diagnostic sufficiency of routine activity-based screening and underscores that normal PC activity cannot exclude a lethal genetic thrombophilia when severe PS deficiency is present.

### 3.3 Broader clinical implications: A call for targeted genetic testing

This case carries immediate implications for diagnosing thrombophilia in young patients, particularly in East Asian populations where *PROC*/*PROS1* defects predominate (4, 5). It advocates for a revised diagnostic algorithm in high-risk, idiopathic PTE that incorporates genetic testing irrespective of standard activity assay results.

Specifically, when a young patient presents with high-risk PTE accompanied by severe PS deficiency (e.g., activity <50%), targeted genetic testing for *PROS1* and *PROC* should be strongly considered even if PC activity is normal. This approach is critical to uncover the “cryptic” synergistic risk demonstrated here, where severe PS deficiency unmasks a pathogenic *PROC* variant.

Identifying such high-risk genotypes is essential for:

Accurate, lifelong risk stratification in the proband, mandating indefinite therapeutic anticoagulation.

Informed cascade screening of first-degree relatives. As exemplified by our case and others (16), asymptomatic carriers can transmit risk alleles; identifying them enables personalized counseling and prophylactic strategies during high-risk periods (e.g., surgery, pregnancy).

Anticipating thrombotic risk beyond the presenting event, as similar genetic synergies may predispose to severe thrombosis at other sites (e.g., cerebral veins) under stress (13).

### 3.4 Management insights and limitations

From a management perspective, this case validates VA-ECMO as a vital bridge to definitive intervention (e.g., thrombectomy) in patients with a genetic thrombophilia predisposition who present with refractory shock (17, 18). The successful outcome underscores the indispensability of a prepared, multidisciplinary team integrating critical care, interventional cardiology/radiology, and hematology expertise.

We acknowledge the inherent limitations of a single case report. Primarily, the proposed synergistic mechanism lacks direct biochemical validation through functional studies. Furthermore, the optimal long-term anticoagulation strategy for patients with this specific high-risk genotype warrants further prospective investigation.

## 4 Patient perspective

The patient and his family expressed immense gratitude for the lifesaving care. The genetic diagnosis provided a pivotal explanation for the catastrophic event, shifting their understanding from a random tragedy to a defined, heritable condition. While acknowledging the complexity of indefinite anticoagulation, they value the clarity it brings for long-term planning. They have actively initiated cascade screening within the family, emphasizing the importance of preventing similar events in relatives. Determined to reclaim his life, the patient adheres strictly to his medication and has incorporated regular daily exercise (e.g., >10,000 steps) as a cornerstone of his health regimen. His goal is to maintain vigilance while pursuing a full and active life.

## 5 Conclusion

This case elucidates a novel “two-hit, unmasking” mechanism in thrombophilia: severe PS deficiency unveils the latent threat of a pathogenic *PROC* mutation despite normal PC activity. This finding challenges the diagnostic sufficiency of standard functional assays and demonstrates that normal PC activity cannot rule out high-risk genetic thrombophilia.

Consequently, we advocate for a revised diagnostic paradigm in young patients with high-risk, idiopathic PTE, with particular urgency in East Asian populations where *PROC*/*PROS1* defects are predominant. Workup should prioritize targeted genetic testing for *PROC* and *PROS1* alongside activity assays, which is essential for accurate diagnosis, guiding lifelong anticoagulation, and enabling preventive family screening.

Finally, the case confirms that a rapid, multidisciplinary approach centered on VA-ECMO as a bridge to definitive intervention is crucial for salvaging patients with such catastrophic, genetically driven thrombosis.

## Data Availability

The datasets presented in this study can be found in online repositories. The names of the repository/repositories and accession number(s) can be found in the article/Supplementary Material.
